# P-512. Assessing the Validity of ICD-9/10 Codes and Microbiologic data for Congenital CMV Diagnosis in an Integrated Health System

**DOI:** 10.1093/ofid/ofaf695.727

**Published:** 2026-01-11

**Authors:** Tara L Greenhow, Gail J Demmler-Harrison, Yinge Qian, Robert Yolken, John D Diaz-Decaro, Colin Kunzweiler, Lisa A Croen

**Affiliations:** Kaiser Permanente Northern California, San Francisco, CA; Baylor College of Medicine, Bellaire, TX; Kaiser Permanente Northern California, San Francisco, CA; Johns Hopkins School of Medicine, Baltimore, Maryland; Moderna, Inc., Potomac, Maryland; Moderna Therapeutics, Inc., Cambridge, Massachusetts; Kaiser Permanente Northern California, San Francisco, CA

## Abstract

**Background:**

Universal screening at birth for cytomegalovirus (CMV) detects congenital CMV (cCMV) in 0.4% to 1% of infants. Accurate coding of cCMV is necessary to identify cCMV in large databases. In a large integrated healthcare delivery system, we aimed to evaluate the accuracy of ICD-9/10 codes and microbiologic data in identifying children with cCMV.

**Table 1:**
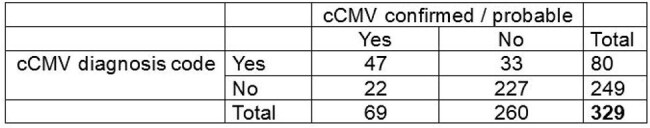
Utility of cCMV diagnosis codes in predicting confirmed or probable cCMV

**Table 2:**
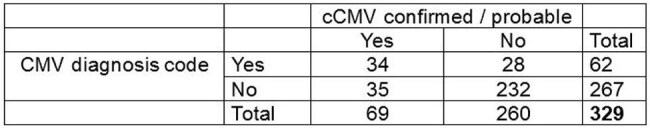
Utility of CMV diagnosis codes in first 45 days of life in predicting confirmed or probable cCMV

**Methods:**

Children born between 2011-2020 with a cCMV diagnosis (ICD-9 771.1, ICD-10 P35.1) in childhood, a CMV diagnosis (ICD-9 078.5, ICD-10 B25) within 45 days of life, or viral testing for CMV under age 2 years were reviewed for clinical, laboratory and radiographic evidence of cCMV infection. Virologic studies included CMV antibody, CMV polymerase chain reaction (PCR) and viral / CMV culture. Children were categorized as cCMV confirmed, probable, unlikely or ruled out.

**Table 3:**
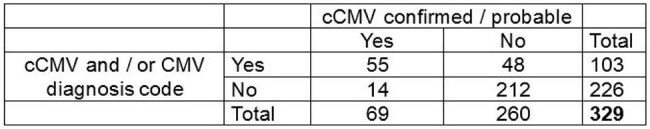
Utility of either cCMV diagnosis codes and / or CMV diagnosis codes in first 45 days of life in predicting confirmed or probable cCMV

**Results:**

In a birth cohort of 401,043, 69 children (0.02%) had confirmed (48) or probable (21) cCMV. Our cohort of 329 children included 80 with a cCMV diagnosis code, 62 with a CMV diagnosis code in the first 45 days of life and 226 with CMV viral testing in the first 2 years of life. Of the 80 children with a cCMV diagnosis code, 47 (59%) had confirmed or probable cCMV (Table 1). Of 22 children with cCMV without a cCMV diagnosis code, 8 had a CMV diagnosis code including 7 with confirmatory CMV PCR in the first 21 days of life and 1 with microcephaly and positive CMV PCR at age 42 days. The 14 children without either a cCMV or CMV diagnosis code had abnormal CMV testing and symptoms of cCMV including microcephaly, sensorineural hearing loss, abnormal brain imaging, and/or, before postnatal day 21, thrombocytopenia or elevated direct bilirubin. The sensitivity and specificity of cCMV diagnosis codes were 68% and 87%, respectively (Table 1). The sensitivity and specificity of CMV diagnosis codes in the first 45 days of life were 49% and 89%, respectively (Table 2). Using either cCMV and/or CMV diagnosis codes the sensitivity was 80% and specificity was 82% (Table 3).

**Conclusion:**

cCMV diagnosis codes appear unreliable: missing 32% of children with cCMV and falsely identifying 13% of children without evidence of cCMV. To improve case ascertainment, cCMV and CMV diagnoses and symptoms recorded in large databases should be confirmed with virologic evidence.

**Disclosures:**

Tara L. Greenhow, MD, Moderna Therapeutics, Inc.: Grant/Research Support Gail J. Demmler-Harrison, MD, Elsevier: Royalties|Merck: Grant/Research Support|Microgen: Advisor/Consultant|Microgen: Grant/Research Support|Moderna: Advisor/Consultant|Moderna: Grant/Research Support|WEB MD Medscape: Honoraria|WEB MD Medscape: travel|Wolters Kluwer UpToDate: Royalties Yinge Qian, PhD, Moderna Therapeutics, Inc.: Grant/Research Support John D. Diaz-Decaro, PhD, MS, ModernaTX, Inc: Stocks/Bonds (Private Company) Colin Kunzweiler, PhD, Moderna Therapeutics, Inc.: Current employee|Moderna Therapeutics, Inc.: Stocks/Bonds (Public Company) Lisa A. Croen, PhD, Moderna Therapeutics, Inc.: Grant/Research Support

